# Immunohistochemical Signature Add Prognostic Value in Patients With Early and Intermediate Hepatocellular Carcinoma Underwent Curative Liver Resection

**DOI:** 10.3389/fonc.2020.616263

**Published:** 2021-01-11

**Authors:** Yannan Bai, Yuane Lian, Xiaoping Chen, Jiayi Wu, Jianlin Lai, Funan Qiu, Songqiang Zhou, Zijing Zhu, Yifeng Tian, Yaodong Wang, Yinghong Yang, Maolin Yan

**Affiliations:** ^1^ Department of Hepatobiliopancreatic Surgery, Fujian Provincial Hospital, Shengli Clinical Medical College of Fujian Medical University, Fuzhou, China; ^2^ Department of Pathology, Fujian Medical University Union Hospital, Fuzhou, China; ^3^ Department of Statistics, College of Mathematics and Informatics & FJKLMAA, Fujian Normal University, Fuzhou, China

**Keywords:** hepatocellular carcinoma, immunohistochemistry, prognostic marker, classifier, prognosis

## Abstract

Hepatocellular carcinoma (HCC) is the third most lethal cancer worldwide; however, accurate prognostic tools are still lacking. We aimed to identify immunohistochemistry (IHC)-based signature as a prognostic classifier to predict recurrence and survival in patients with HCC at Barcelona Clinic Liver Cancer (BCLC) early- and immediate-stage. In total, 567 patients who underwent curative liver resection at two independent centers were enrolled. The least absolute shrinkage and selection operator regression model was used to identify significant IHC features, and penalized Cox regression was used to further narrow down the features in the training cohort (n = 201). The candidate IHC features were validated in internal (n = 101) and external validation cohorts (n = 265). Three IHC features, hepatocyte paraffin antigen 1, CD34, and Ki-67, were identified as candidate predictors for recurrence-free survival (RFS), and were used to categorize patients into low- and high-risk recurrence groups in the training cohort (P < 0.001). The discriminative performance of the 3-IHC_based classifier was validated using internal and external cohorts (P < 0.001). Furthermore, we developed a 3-IHC_based nomogram integrating the BCLC stage, microvascular invasion, and 3-IHC_based classifier to predict 2- and 5-year RFS in the training cohort; this nomogram exhibited acceptable area under the curve values for the training, internal validation, and external validation cohorts (2-year: 0.817, 0.787, and 0.810; 5-year: 0.726, 0.662, and 0.715; respectively). The newly developed 3-IHC_based classifier can effectively predict recurrence and survival in patients with early- and intermediate-stage HCC after curative liver resection.

## Introduction

Hepatocellular carcinoma (HCC) is the third most lethal cancer worldwide, with 780,000 annual deaths recorded globally (1). Although hepatic resection remains the treatment of choice to achieve a cure in patients with HCC, a high recurrence rate after curative resection is still a major cause of death (2–4). Conventional HCC staging systems, such as the Barcelona Clinic Liver Cancer (BCLC) (5), Japan Integrated Staging (JIS) (6), and Tumor-Node-Metastasis (TNM) systems (7), use conventional clinicopathological features (liver function, tumor size, number, and vascular invasion) for prognostic stratification. However, HCC is a heterogeneous entity, with considerable variation in clinical outcomes, even for identical tumor stages. There is an ongoing pursuit for prognostic biomarkers for cancer. Clinicopathological parameters, and integrative and comprehensive genomic alterations have been analyzed and used to stratify patients into various groups with different prognoses (8–18), none of these stratification tools have been routinely employed in staging systems for predicting recurrence and survival after surgical resection.

As an inexpensive and easy-to-use pathological technique, immunohistochemistry (IHC) is routinely used for analyzing HCC carcinogenesis, development, and invasiveness. Despite the development of IHC markers with diagnostic value, prognostic markers are not well-established. Previous studies have investigated the value of IHC-based classifiers as predictors of overall survival (OS), but not of recurrence-free survival (RFS) (10, 19). It should be noted that OS is mainly determined by the status of liver function and post-recurrence treatment, yet RFS reflects the biological heterogeneity of HCC. A panel of common IHC markers was selected—including hepatocyte markers hepatocyte paraffin antigen 1 (HepPar-1) and Glypian-3, cytokeratin (CK) proteins CK18 and CK19, angiogenesis-related CD34, canalicular staining marker CD10, epithelial-mesenchymal transition markers vimentin (VIM), melanocyte marker HMB45, and tumor proliferation and aggressiveness marker Ki-67—based on its diagnostic value with respect to carcinogenesis, development, and invasiveness of HCC. We hypothesize that a combination of IHC markers could have greater prognostic value than each of the markers alone when considering tumor recurrence and long-term survival.

The aim of this study was to develop a recurrence-related IHC-based classifier. We used a cohort of 201 patients with HCC after curative liver resection using the least absolute shrinkage and selection operator (LASSO) Cox regression model and validated the classifier using an internal, and an external cohort of 101, and 265 patients, respectively. We then developed a prognostic nomogram incorporating the BCLC stage, microvascular invasion (MVI), and the 3-IHC_based classifier to improve the predictive power. This study may contribute to early detection of recurrence in patients with HCC who underwent curative resection, thereby possibly improving patient outcome.

## Methods

### Patients and Samples

Between March 2010 and December 2014, a total of 1,436 consecutive patients who had undergone curative-intent liver resection for HCC from two tertiary Chinese centers were retrospectively screened. The following inclusion criteria were used: HCC at BCLC early- or intermediate-stage, without extrahepatic metastasis or other homochromous malignancies, and without any anticancer treatment before surgery. A total of 302 HCC patients during surgical resection were included at Fujian Provincial Hospital (FPH) and 265 patients at Fujian Medical University Union Hospital (FMUUH). Patients were excluded when the tumor specimen or clinicopathological data were missing. This retrospective study was approved by the Institutional Review Board of FPH and FMUUH and was performed in accordance with the Declaration of Helsinki. Written informed consent for tissue collection was obtained from each patient prior to the study. Patients at FPH were further randomly stratified into a training cohort (201 patients) and an internal validation cohort (101 patients) at a 2:1 ratio.

The data were censored on December 31, 2019. Patients were followed up at 2-month intervals in the first year after surgery and at 3-month intervals thereafter. The clinicopathological data are presented in **Table 1**. The computation of Child-Turcotte-Pugh (CTP) functional class, albumin-bilirubin (ALBI) grade, and BCLC stage were determined as per standard published methodologies (5, 20). The severity of liver fibrosis and tumor differentiation was defined using the Ishak scoring system and Edmondson grading system, respectively (21, 22). MVI was defined as the presence of tumor emboli in a portal vein, hepatic vein, or within a vascular space lined by endothelial cells that was visible only on microscopy (23). The primary clinical endpoints were RFS, calculated from the date of resection to the date of recurrence, metastasis, or last follow-up, and OS, calculated from the date of resection to the date of death or last follow-up.

**Table 1:** Clinicopathological factors of patients in training and validation cohort.

Baseline characteristics	Number/Median [IQR]*		
	Internal cohort (n = 201) (n = 101)	Training cohort	External cohort (n = 265)
Gender			
M: F	175: 26	87: 14	233: 32
Age (year)	61 [50, 68]	59 [51, 67]	56 [48, 62]
≥60: <60	111: 90	49:52	93: 172
HBsAg			
Pos: Neg	169: 32	85: 32	239: 26
**HCV**			
Pos: Neg	**9:192**	**6:95**	**11:254**
ALB (g/l)	43.9 [40.9, 46.6]	44.4 [40.7, 46.7]	40.2 [37.8, 43.0]
TB (umol/l)	13.8 [10.8, 18.7]	14.0 [11.0, 18.1]	13.2 [9.6, 17.4]
ALT (U/l)	37 [27, 50]	37 [25, 53]	32 [22, 44]
AST (U/l)	34 [26, 50]	31 [25, 46]	29 [23, 41]
plt (10^(9)/l)	175 [133, 231]	175 [137, 223]	161 [127, 197]
AFP (ug/l)			
<20: ≥20	91: 110	50: 51	111: 154
CTP score			
A5: A6: B7	169: 22: 10	84: 13: 4	233: 27: 5
ALBI	-2.96 [-3.23, -2.70]	-3.01 [-3.24, -2.65]	-2.69 [-2.92, -2.46]
Size (cm)	4.0[3.0, 8.0]	4.5[3.0, 7.0]	4.0[2.8, 5.5]
Number			
1: 2-3: >3	173: 22: 6	88: 11: 2	218: 38: 9
MVI			
Abs: Pre	149: 52	72: 29	216: 49
Ishak classification			
F0: F1	51: 150	24: 77	63: 202
Grade			
I-II: III-IV	168: 33	85: 16	98: 167
BCLC classification			
early: intermediate	117: 84	59: 42	166: 99
3-IHC-based classifier			
Low: high	156: 45	79: 22	182: 83
RFS (month)	42[12, 55]	41[16, 59]	48[20, 60]
OS (month)	55[43, 68]	53[39, 67]	53[41, 62]

M, male; F, female; HbsAg, hepatitis B virus surface antigen; AFP, α-fetoprotein; CTP, Child-Turcotte-Pugh score; ALBI, albumin-bilirubin; MVI, microvascular invasion; F0: Ishak F0-F4; F1: Ishak F5-F6; BCLC, Barcelona Clinic Liver Cancer; IHC, Immunohistochemistry; RFS, recurrence-free survival; OS, overall survival; Pos, positive; Neg, negative; Abs, absence; Pre, presence; IQR, interquartile range.

*Median with interquartile range are shown for quantitative variables, whereas counts are shown for categorical variables.

### Immunohistochemistry

Formalin-fixed and paraffin-embedded tissue sections (5-µm thick) were obtained. Rabbit monoclonal anti-bodies against HepPar-1, Glypian-3, CK18, CK19, CD10, CD34, VIM, HMB45, and Ki-67 were used (MXB Biotechnologies, Inc., Fuzhou, China). The IHC outcomes for each marker were semiquantitative evaluated by two independent and trained pathologists who were blind to the clinical outcomes (LY AND YY). HepPar-1, Glypian-3, CK18, CK19, CD10, CD34, VIM, and HMB45 were localized in the cytoplasm of HCC cells, while Ki-67 was localized in the nuclei. The immunoreaction was recorded as the percentage of positively stained cells and cell staining intensity (absent, weak, moderate or strong) in 3 respective areas at ×200 magnification, and the mean value was adopted. Dichotomization as negative (absent/weak staining) or positive (moderate/strong staining) was then determined based on the reactivity of IHC markers (10, 24). Representative expressions of HepPar-1, CD34 and Ki-67 are shown in **Figure S1**.

### Statistical Analysis

Initially, a LASSO Cox regression model with penalty parameter tuning (10-fold cross-validation) was used to identify the most useful prognostic IHC-based markers. An L1 penalized Cox analysis was performed to further narrow down markers in the training cohort. A multi-marker classifier, derived from the prognostic score for each selected marker, was constructed based on RFS. Cumulative OS and RFS were evaluated using the Kaplan–Meier method and compared using the log-rank test, followed by multivariate Cox regression to identify significant variables. A nomogram was constructed based on the results of the Cox regression models. A decision curve analysis (DCA) was used to assess the clinical utility of the nomogram.

All statistical analyses were performed using R version 3.6.1 with the packages glmnet, pROC, and rms. A two-sided P-value <0.05 was considered significant.

## Results

### Patient Characteristics

**Figure 1:**
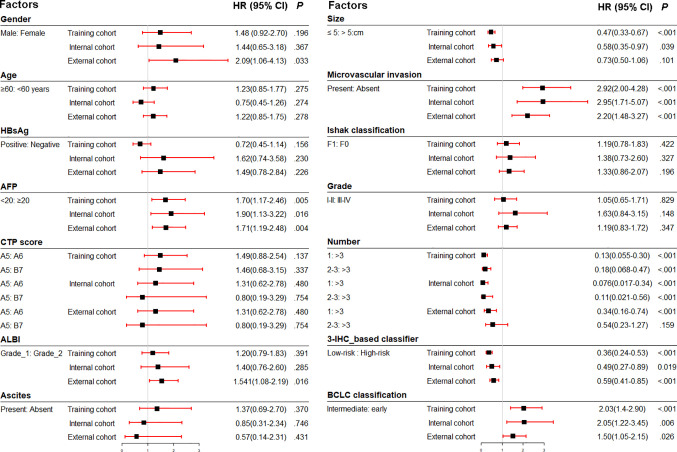
Association of clinicopathologic characteristics with recurrence-free survival in three cohorts. HBsAg, hepatitis B virus surface antigen; AFP, α-fetoprotein; ALBI, albumin-bilirubin; CTP, Child-Turcotte-Pugh score; IHC, immunohistochemistry; BCLC, Barcelona Clinic Liver Cancer; HR, hazard ratio.

A total of 567 patients was included in the study, including 201 in the training cohort, 101 in the internal validation cohort, and 265 in the external validation cohort. Characteristics of the studied populations in the training, internal validation, and external validation cohorts are presented in **Table 1** and **Figure 1**. The median follow-up time for 567 patients with HCC was 53.0 months (range, 3.0 to 84.0 months). During the follow-up, 53.6% of the patients (304 of 567) exhibited recurrence, and 33.9% of the patients (192 of 567) died. For patients who had not been diagnosed with HCC recurrence, adjuvant transarterial chemoembolization (TACE), at the interval of 2 months from surgery, was routinely recommended for patients with high risk factors (tumor size larger than 5 cm, multiple tumors, MVI, poor-differentiation grade, and so on) after completely informing the potential benefits and risks of this treatment. Among 304 patients with recurrence, 107 (35.2%) underwent a repeated resection or ablation, 92 (30.3%) received TACE, 51 (16.8%) received targeted therapy, and 54 (17.8%) received no further treatment. For the entire cohort, the 2- and 5- year RFS rates were 66.5% and 48.7%, respectively, and the 2- and 5-year OS rates were 85.0% and 69.0%, respectively.

### Feature Selection and Predictive Immunohistochemistry-Based Signature Building

**Figure 2:**
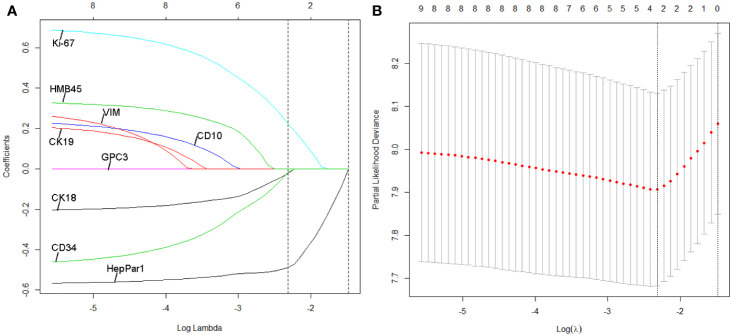
Least absolute shrinkage and selection operator (LASSO) coefficient profiles of the three selected IHC signatures. **(A)** Tuning parameter (selection by 10-fold cross-validation *via* minimum criteria. Partial likelihood deviance was plotted versus log(Lamda). **(B)** Coefficient profile of the IHC markers associated with recurrence-free survival (RFS) of patients with HCC patients at early- and intermediate-stage. Vertical line is shown at the optimal value with three nonzero coefficients.

**Figure 3:**
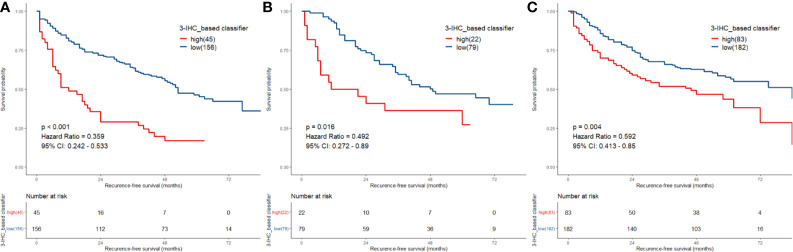
Comparison of recurrence-free survival (RFS) in low-risk vs. high-risk patients stratified by 3-IHC_based classifier. **(A)** Training cohort; **(B)** internal validation cohort; **(C)** external validation cohort.

We identified potential IHC features for RFS prediction from among a set of markers (HepPar-1, Glypian-3, CK18, CK19, CD10, CD34, VIM, HMB45, and Ki-67) using the LASSO Cox regression model. The nine features were reduced to three prognostic markers (HepPar-1, CD34, and Ki-67) in the training cohort and features with penalized Cox coefficients were included in the regression model (**Figure 2**). To better understand the performance of the IHC signature for predicting recurrence, a 3-IHC_based risk score for each patient was derived as follows: risk score = (0.7280 × Ki-67) - (0.4495 × CD34) - (0.6027 × HepPar-1). The status of each IHC marker was categorized as negative (equals 0) and positive (equals 1). The optimum cut-off value for the risk score was defined as −0.613 based on the training cohort. We assigned patients with a risk score exceeding −0.613 to the high-risk group (n = 45) and others to the low-risk group (n = 156). On applying the cutoff, the low-risk group had a better RFS (hazard ratio [HR], 0.359; 95% CI, 0.242–0.533; P < 0.001; **Figure 3A**) than the high-risk group in the training cohort.

### Validation of the Predictive Immunohistochemistry Signature

To assess the robustness of the 3-IHC_based classifier, validation analyses were performed using in both the internal and external validation cohorts. In the internal validation cohort, the 3-IHC_based classifier categorized 79 patients (78.2%) into the low-risk group and 22 patients (21.8%) into the high-risk group with a significant difference in RFS (HR, 0.492; 95% CI, 0.272–0.890; P = 0.016; **Figure 3B**). Similar analyses indicated that 182 low-risk patients (68.7%) had a better RFS than 83 high-risk patients (31.3%) in the external validation cohort (HR, 0.592; 95% CI, 0.413–0.850; P = 0.004; **Figure 3C**). In the combined cohort containing 567 patients, recurrence occurred at a later time point in the low-risk group than in the high-risk group (median time, 22.0 [range, 1.0–84.0] versus 9.0 [range, 1.0–84.0] months; P < 0.001; **Figure S2**).

We also investigated the performance of the 3-IHC_based classifier for predicting OS and effectively stratified patients into low- and high-risk groups with respect to long-term prognosis in all three cohorts (training cohort: HR, 0.362; 95% CI, 0.222–0.592; P < 0.001; internal validation cohort: HR, 0.471; 95% CI, 0.224–0.991; P = 0.042; external validation: HR, 0.427; 95% CI, 0.281–0.650; P < 0.001; **Figure S3**). Upon setting 2 years post-hepatectomy as the threshold for early recurrence (ER, representing true recurrence from the primary HCC) or late recurrence of HCC (LR, representing *de novo* HCC recurrence) (25), we assigned 304 patients with recurrence to an ER group (190 patients) and an LR group (114 patients). In our study, the 3-IHC_based classifier maintained its discriminative ability for predicting OS in patients with ER (HR, 0.717; 95% CI, 0.517–0.996; P = 0.047) but not for patients with LR (**Figure S4**).

**Figure 4:**
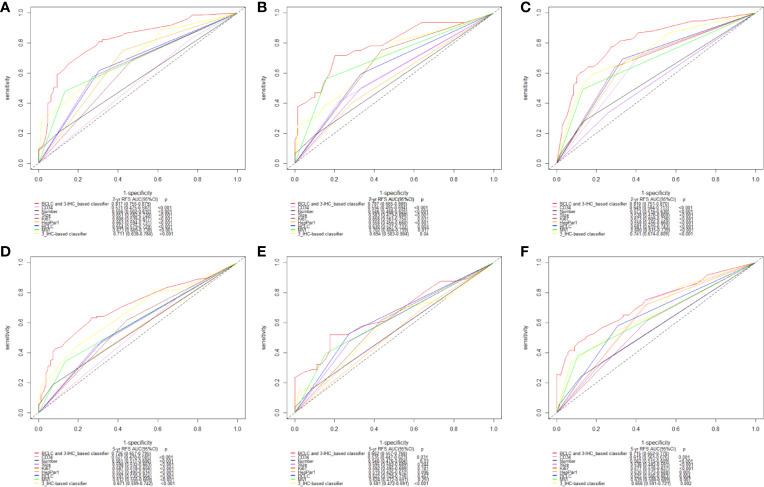
Time-dependent receiver operating characteristic (ROC) analysis on basis of recurrence-free survival (RFS) [2-year, **(A–C)**; and 5-year, **(D–F)**; respectively] was used to compare performance of 3-IHC_based classifier with the clinicopathological factors and three single immunohistochemistry (IHC) features alone. Training cohort **(A, D)**; Internal validation cohort **(B, E)**; External validation cohort **(C, F)**. BCLC, Barcelona Clinic Liver Cancer; MVI, microvascular invasion.

Furthermore, the Area Under the Receiver Operating Characteristics (AUC) curve analysis for the 3-IHC_based classifier was performed. As shown in **Figure 4**, the AUC values of the 3-IHC_based classifier for 2- and 5-year RFS prediction in the training cohort (0.711, 95% CI: 0.638–0.784; 0.671, 95% CI: 0.599–0.742; respectively) indicated significantly better discrimination ability than that of the individual IHC markers. Similar discrimination ability was observed in case of the internal (AUC for 2- and 5-year RFS, 0.694, 95% CI: 0.583–0.804; 0.581, 95% CI: 0.472–0.691) and external validation cohort (0.741, 95% CI: 0.674–0.809; 0.656, 95% CI: 0.591–0.721). Moreover, AUC value for the combination of the BCLC stage and 3-IHC_based classifier exhibited better performance for predicting RFS than those for the BCLC stage alone (2-year: 0.817, 0.787, and 0.810; 5-year: 0.726, 0.662, and 0.715; P < 0.001, for the training, internal validation, and external validation cohorts, respectively), indicating that the 3-IHC_based classifier has better prognostic value than that of the conventional BCLC stage for predicting RFS in HCC (**Figure 4**).

### Nomogram Construction and Clinical Usage

**Figure 5:**
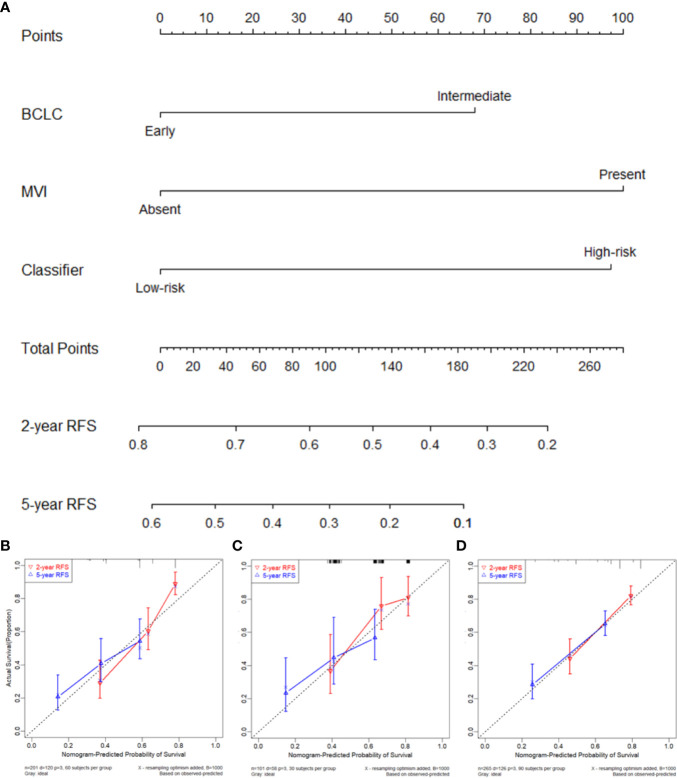
**(A)** Nomogram to predict the 2- and 5-year recurrence-free survival (RFS). Calibration curve for RFS nomogram in training cohort **(B)**, internal validation cohort **(C)**, and external validation cohort **(D)**.

Prior to the development of a clinically useful prognostic algorithm for predicting individual recurrence probabilities, we evaluated common clinicopathological features by multivariate Cox regression analyses based on RFS using a combination of significant variables and 3-IHC_based classifier (**Figure 1** and **Table 2**). Based on the significant predictors in multivariate analysis (BCLC stage, MVI, and 3-IHC_based classifier), we constructed a nomogram to predict 2- or 5-year RFS in the training cohort (**Figure 5A**). The Calibration curves showed good correspondence between the predicted and actual probability of 2- or 5-year RFS for all three cohorts (**Figures 5B–D**). Furthermore, a DCA was used to compare the 3-IHC_based nomogram and each predictor alone (BCLC stage, MVI, and 3-IHC_based classifier, respectively). DCA graphically showed that the 3-IHC_based nomogram provided a net benefit over the range of threshold probabilities for 2- or 5-year RFS when compared with each individual predictor (**Figure S5**).

**Table 2:** Multivariate Cox Analysis of Clinicopathologic Factors and 3_IHC-based classifier with Recurrence-Free Survival in the Entire Cohort.

Factor	Multivariate Analysis	
	HR (95% CI)	P
AFP (<20/≥20 ug/l)	1.272 (0.846-1.913)	0.248
BCLC (intermediate/early)	1.723 (1.193-2.487)	0.004
MVI (present/absent)	2.179 (1.454-3.266)	<0.001
3_IHC-based classifier (low-risk/high-risk)	0.512 (0.327-0.799)	0.003

AFP, α-fetoprotein; MVI, microvascular invasion; BCLC, Barcelona Clinic Liver Cancer; IHC, Immunohistochemistry.

## Discussion

In this study, we used IHC to screen the expression status of routinely available markers in resected HCC samples from an FPH cohort. By using the LASSO Cox regression algorithm, we reduced the features to a set of three candidates and developed an IHC signature (i.e., a 3-IHC_based classifier) in the training cohort, which was then validated using internal and external validation cohorts. Based on the classifier, patients with HCC could be stratified into two distinct subgroups with low and high probabilities of recurrence and OS. Interestingly, nearly half of the patients in the high-risk group experienced ER, while recurrent HCC was observed in less than one-quarter in the low-risk group (**Data Supplement**), suggesting that patients in the high-risk group require more intensive medical surveillance within 2 years after curative liver resection. Furthermore, among patients with ER, the 3-IHC_based classifier was able to discriminate between patients with different OS, suggesting the practical predictive value of our classifier in ER entity, which was considered as true recurrence from the primary HCC (25). Finally, to improve management decisions for individualized follow-up and treatment strategies, we integrated the BCLC stage and MVI into a prognostic nomogram to predict the 2- and 5-year RFS in patients with HCC after curative liver resection. Calibration curves showed a good consistency between the predicted and actual RFS. The DCA further supported the prognostic value of the nomogram for clinical application.

In our study, we developed a 3-IHC_based classifier based on IHC markers that are routinely used in clinical pathology. Each candidate IHC marker has an established biological and diagnostic role in the carcinogenesis, development, and invasiveness of HCC. HepPar-1 and Glypian-3 are hepatocyte functional markers associated with the degree of tumor differentiation (with different sensitivities) (26). CK18 and CK19 are widely used to distinguish between HCC and biliary-derived carcinoma (27). CD34 is used to determine the degree of tumor angiogenesis (28). CD10 and HMB45 are used for the differential diagnosis of HCC from exogenous metastases and other liver mesenchymal tumors (29, 30). VIM is a marker of tumor epithelial-mesenchymal transition and HCC metastasis (31); and Ki-67 is a marker of proliferation (32). It should be noted that some of these IHC markers or combinations of markers have been identified as risk factors with prognostic value for predicting recurrence or long-term survival in HCC after surgery. For example, High-level Ki-67 expression in HCC tumor was associated with more rapid ER (32). HepPar-1, as a hepatocyte specific antigen, its data of prognostic significance in HCC was limited and inconsistent. However, combination with CK19 might increase the prognostic power for predicting OS in HCC (33). A previous study developed a morpho-molecular prognosticator of patients with HCC based on the combination of several clinicopathological features and IHC markers (10). The prognosticator was able to classify subgroups with different OS and RFS, however, risk score for predicting RFS was identical to the one for predicting OS which calculated using Cox regression model based on OS outcomes. In fact, only two IHC features (P53 and CD31) were identified as independent risk factors for tumor recurrence. Recently, another study proposed a prognostic and a recurrent classifier separately to predict OS and RFS for patients with HCC based on a set of 29 IHC features (19). The study showed a favorable prognostic model with high prediction accuracy for 3-year recurrence in all three cohorts (95% CI: 0.734, 0.693–0.710; 0.749, 95% CI: 0.677–0.812; 0.730, 95% CI: 0.635–0.812; respectively). The AUC value for the prognostic classifier developed was slightly lower than the one developed in our study when considering 2-year recurrence (0.817, 95% CI: 0.755–0.879; 0.787, 95% CI: 0.685–0.888; and 0.810, 0.751–0.870; respectively) and 5-year recurrence in all three cohorts (0.726, 95% CI: 0.657–0.795; 0.662, 95% CI: 0.557–0.768; 0.715, 95% CI: 0.652–0.778; respectively). Besides, MVI was not included and assessed as an important clinicopathological feature. To further improve the prognostic accuracy and to identify the most effective IHC signature, nine markers were reduced to three prognostic features using the LASSO method for variable selection in the Cox model (34). As indicated in our study, the proposed signature showed substantial prognostic ability with a higher AUC value than that of predictors evaluated in previous studies.

Several strengths of this study should be noted. First, a 3-IHC_based signature was identified as prognostic classifier to stratify HCC risk groups based on the recurrence probability. This is important because RFS is a more accurate representation of the biological characteristics of HCC than OS, which is mainly influenced by liver function and post-recurrence treatment. Second, compared with previous studies—based on high-throughput genetic profiles—we focused on clinically available IHC-based markers, which are characterized by the following features: potential for easy, inexpensive, and reliable results. These pathological diagnosis-based markers are expected to have substantial prognostic value. Third, patients with advanced HCC were not included in our study to exclude highly unfavorable factors, such as macrovascular invasion and end-stage liver failure. We included patients classified as BCLC early-stage and intermediate-stage and validated the features in samples from two-centers, considering that the classifications and therapeutic modalities for BCLC early- and intermediate-stage HCC remain controversial (2–6).

Although the 3-IHC_based nomogram showed substantial power for tumor recurrence and survival in patients with HCC after curative liver resection, several limitations should be noted. First, we used retrospective data from two centers. Second, only nine IHC markers were assessed in this study. This might explain the failure of the 3-IHC_based classifier in stratifying patients with LR based on OS. The assessment of additional IHC markers related to cell cycle regulation, angiogenesis, invasiveness, immunoreactivity, tumor microenvironment, etc., will provide more comprehensive data in the future. Third, prospective studies using multiple cohorts are required to verify our findings.

In summary, our study shows that the newly developed 3-IHC_based classifier is a feasible prognostic tool for predicting recurrence and survival in patients with HCC classified as BCLC early- and intermediate-stage after curative liver resection. By integrating the classifier with the BCLC stage and MVI, the nomogram might improve personized prognostic assessments and aid in management decisions and development of treatment strategies.

## Data Availability Statement

The raw data supporting the conclusions of this article will be made available by the authors, without undue reservation.

## Ethics Statement

The studies involving human participants were reviewed and approved by the Institutional Review Board of Fujian Provincial Hospital and Fujian Medical University Union Hospital. The patients/participants provided their written informed consent to participate in this study.

## Author Contributions

BY and LY conceptualized the study. CX and ZZ contributed to the methodology. WJ, LJ, QF, ZS, and TY contributed to the data curation. BY and LY wrote and prepared the the original draft. WY, YY, and YM wrote, reviewed, and edited the manuscript. BY, LY, and CX contributed to the project administration. All authors contributed to the article and approved the submitted version.

## Funding

Research reported in this publication was supported in part by the Foundation for Medical Youth Program of Fujian Province (Grant Number: 2014-1-5), Foundation for Youth Program of Fujian Provincial Hospital (Grant Number: 2014YNQN072), Foundation for Medical Innovation of Fujian Province (Grant number: 2015-CX-5), Fujian Provincial Natural Science Foundation (Grant number: 2017J01177), and high-level hospital foster grants from Fujian Provincial Hospital, Fujian province, China (Grant number: 2019HSJJ03).

## Conflict of Interest

The authors declare that the research was conducted in the absence of any commercial or financial relationships that could be construed as a potential conflict of interest.
