# Supplementary material for: Immunohistochemical Signature Add Prognostic Value in Patients With Early and Intermediate Hepatocellular Carcinoma Underwent Curative Liver Resection
Source: Front Oncol. 2021 Jan 11;10:616263. doi: 10.3389/fonc.2020.616263 (PMC7874098; doi:10.3389/fonc.2020.616263)

**Data Supplement**

Figure S1, Represented IHC staining of HepPar1 (A, D), CD34 (B, E), and Ki-67 (C, F). Upper panel, negative IHC staining; low panel, positive IHC staining.

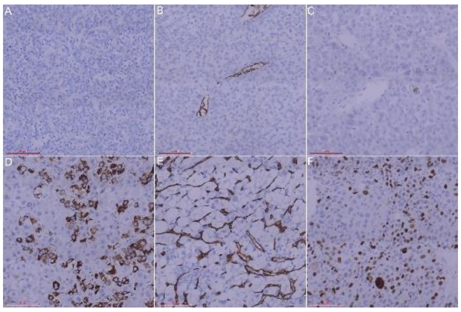


Figure S2, Comparison of RFS in low-risk vs. high-risk patients in combined cohort of training and validation cohorts.

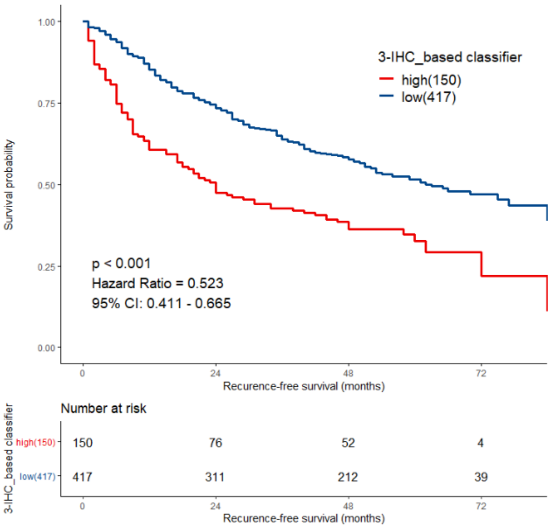


Figure S3, Comparison of OS in low-risk vs. high-risk patients stratified by 3-IHC_based classifier. A, Training cohort; B, internal validation cohort; C, external validation cohort.

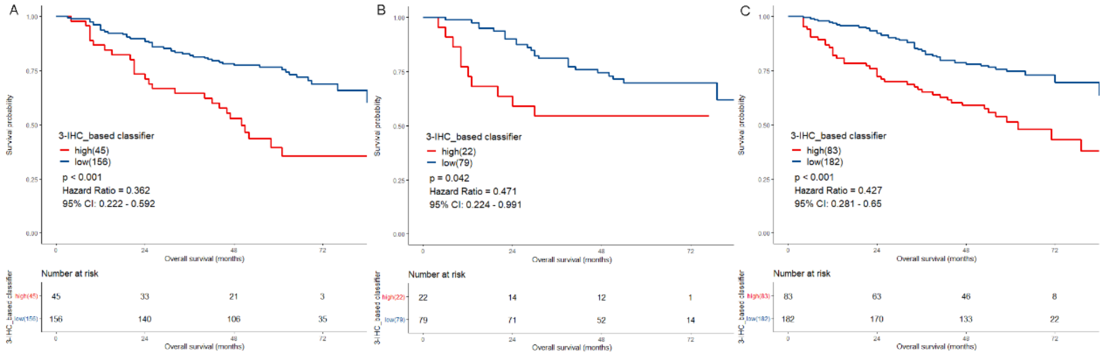


Figure S4, Comparison of OS in low-risk vs. high-risk patients stratified by 3-IHC_based classifier. A, ER group (n = 190), which is mainly from true recurrence, and B, LR group (n = 114), which is usually recognized as de novo cancer.

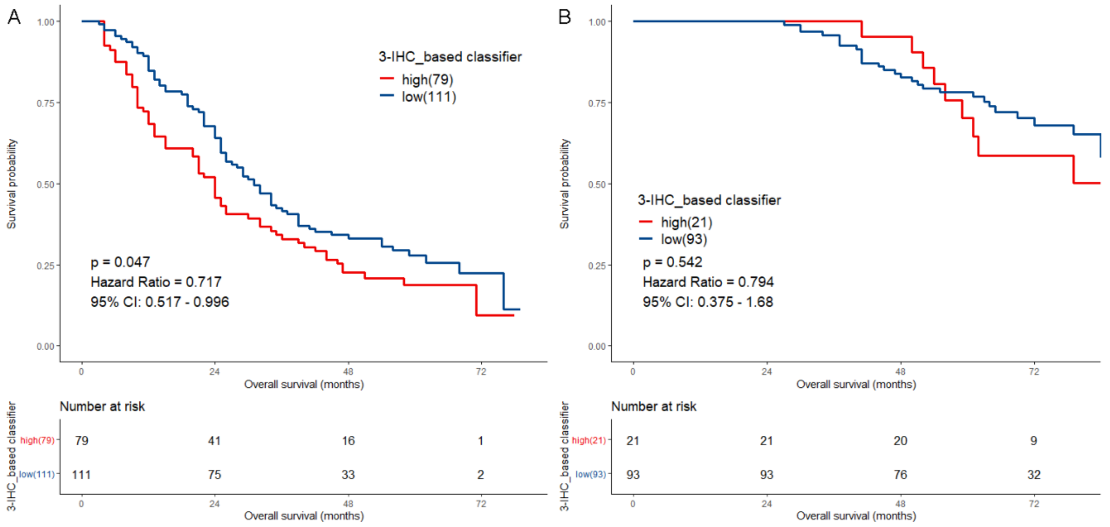


Figure S5, Decision curve analysis (DCA) was used to evaluate clinical net benefit between the nomogram and BCLC staging and MVI, in terms of RFS (2-yr, A-C; 5-yr, D-F; respectively). The y-axis measures the net benefit. The he horizontal solid black line assumed no patients would relapse, and the solid red line assumed all patients would relapse.

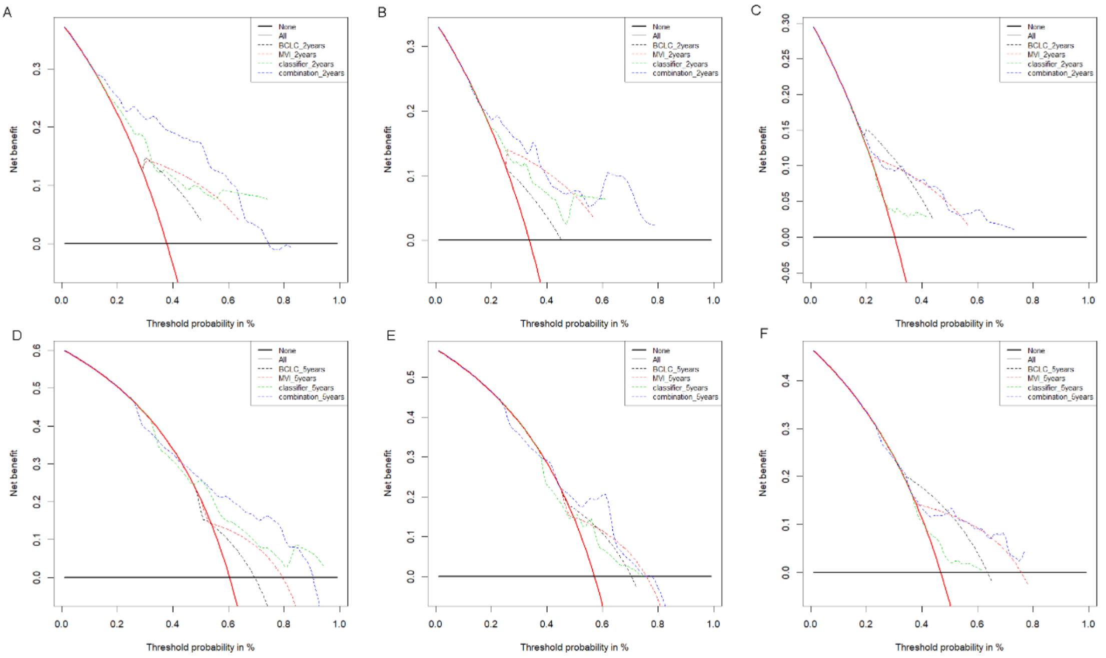

Supplement: Supplementary file 1 [file DataSheet_1.pdf]
